# Broadband Epsilon-Near-Zero Perfect Absorption in the Near-Infrared

**DOI:** 10.1038/srep12788

**Published:** 2015-08-04

**Authors:** Junho Yoon, Ming Zhou, Md. Alamgir Badsha, Tae Young Kim, Young Chul Jun, Chang Kwon Hwangbo

**Affiliations:** 1 Department of Physics, Inha University, Incheon 402-751, Republic of Korea; 2 School of Materials Science and Engineering, Ulsan National Institute of Science and Technology (UNIST), Ulsan 689-798, Republic of Korea

## Abstract

Perfect absorption (PA) of incident light is important for both fundamental light-matter interaction studies and practical device applications. PA studies so far have mainly used resonant nanostructures that require delicate structural patterning. Here, we realize tunable and broadband PA in the near-infrared region using relatively simple thin film coatings. We adjust the growth condition of an ITO film and control its epsilon-near-zero (ENZ) wavelength. We show that this results in highly tunable PA in the telecommunication window. Then, using an ITO multilayer of different ENZ wavelengths, we demonstrate broadband PA that covers a wide range of near-infrared wavelengths. The use of ENZ coatings makes PA adjustable during the film growth and does not require any structural patterning afterward. It also facilitates the chip-scale integration of perfect absorbers with other device components. Broadband PA relaxes the single wavelength condition in previous PA studies, and thus it is suitable for many practical device applications, including sensors, photodetectors, and energy harvesting devices.

Perfect absorption (PA) has attracted much attention for both fundamental studies and device applications^1^,^2^,^3^,^4^,^5^,^6^,^7^,^8^,^9^,^10^,^11^. Total absorption of incident light in a deep subwavelength volume is an interesting research topic itself from a fundamental point of view. This can also lead to highly efficient energy conversion, which is important for many device applications. PA has been studied in various contexts – e.g. in the study of critical coupling^12^,^13^,^14^,^15^,^16^,^17^ and coherent perfect absorption (CPA)^18^,^19^,^20^,^21^,^22^,^23^. Under certain circumstances, incident light can be critically coupled to resonant modes. Or, equivalently, transmission (*T*) and reflection (*R*) can be completely suppressed by destructive interference, resulting in total absorption (*A*) of light (i.e. *A* = 1 − *R* − *T* = 1 when *T* = *R* = 0).

So far, most of these PA studies involved resonant nanostructures that require delicate structural patterning. Recently, it was pointed out that PA can be also achieved in epsilon-near-zero (ENZ) metamaterial structures^24^,^25^. Shortly later, this work was extended to natural plasmonic materials like semiconductors and conducting oxides^26^,^27^ where dielectric constants are tunable by doping densities. In these materials, the real part of the dielectric constant (*Re*[ε]) becomes zero at a certain wavelength (i.e. ENZ wavelength), depending on the doping level. At an ENZ wavelength the normal component (E_Z_) of the electric field in a plasmonic subwavelength thin film becomes very strong, and this can lead to very large light absorption in the film^27^. The maximum absorptance of a free-standing thin film is limited to *A* = 0.5, but it is possible to increase it up to *A* = 1 (i.e. PA) in a proper condition. For example, when a subwavelength plasmonic film is coated on a metallic substrate or the attenuated total reflection (ATR) configuration is employed, the destructive interference condition of reflected light in the transverse magnetic (TM) mode (or the so-called critical coupling condition) can be approximately written as the following equation^27^,
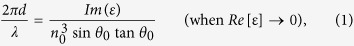
where λ is the wavelength of incident light, *θ*_0_ is the incidence angle, *n*_0_ is the refractive index of the incidence medium, *d* is the film thickness, and ε is the dielectric constant of the film. Note that, in this case, transmission is naturally suppressed due to the opaque substrate or the evanescent wave in the ATR, so we only need to consider the destructive interference of reflected light. Contrary to conventional cases, PA can be achieved in an ultra-thin 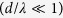, flat film even with a very low optical loss (*Im*[ε]). However, Eq. (1) can be satisfied only at a single wavelength for a given film thickness and incidence angle, and thus it is limited in practical use.

In this paper, we demonstrate tunable and broadband PA using Indium Tin Oxide (ITO) thin films in the ATR configuration. ITO has attracted attention recently as a tunable, near-infrared (IR) plasmonic material^28^,^29^,^30^,^31^,^32^. The ENZ wavelength of an ITO film occurs in the near-IR region, and can be tunable by the film growth condition. Therefore, ITO works as a tunable ENZ material too. Here, using ITO thin films, we adjust PA wavelengths without any structural patterning. Moreover, by depositing an ITO multilayer of different ENZ wavelengths, we experimentally implement broadband PA in the near-IR region. Broadband PA relaxes tight wavelength and incident angle dependencies in previous PA studies, and therefore it is more suitable for many device applications that require tunable and/or broadband light absorption and energy conversion.

## Experimentals

### Film Preparation

For tunable PA we prepared three different ITO films (I1, I2, I3) by altering the film growth condition. First, ITO films were deposited on glass substrates (B270) by RF magnetron sputtering, using a ceramic target of 90 wt % In_2_O_3_ and 10 wt % SnO_2_^33^,^34^. The base pressure in the sputtering chamber was 3.0 × 10^−6^ Torr, and an Ar gas was admitted at 50 sccm until a pressure of 5.5 × 10^−3^ Torr was obtained. The RF power was 30 W and the substrate temperature was ambient. Then, the films were annealed at 250 °C in vacuum for two hours to improve the film quality. We used different vacuum levels in the annealing process for three samples to tailor their optical response: 5 × 10^−6^ Torr (I1), 5 × 10^−3^ Torr (I2), 5 × 10^−1^ Torr (I3). The thicknesses of three films were measured to be 65 nm (I1), 74 nm (I2), and 84 nm (I3).

To implement broadband PA, we deposited a multilayer of ITO films of different doping densities, based on our tunable ITO films studied. The first ITO layer was deposited on a glass substrate (B270) as before, and annealed at 300 °C for 2 hours with a pressure of 10^−6^ Torr. Then, the second ITO layer was deposited on top of it and annealed at 200 °C for 1 hour at 10^−3^ Torr. During the second annealing process, we expect an intermixed layer formed between two ITO layers.

### Film Characterization

**Figure 1:**
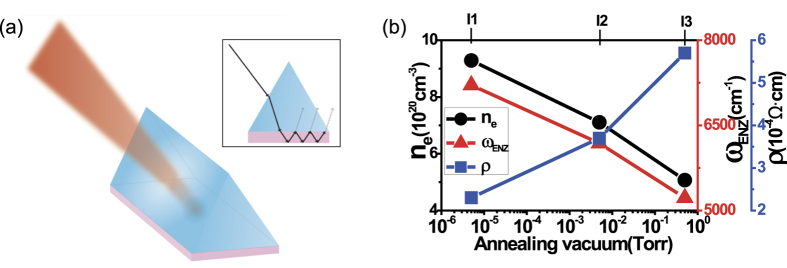
(**a**) Schematic of broadband perfect absorption (PA) experiments. By working in the attenuated total reflection (ATR) configuration, we can suppress transmission and achieve PA in a thin film by finding the destructive interference condition in reflection (e.g. see the inset). (**b**) Free carrier density, ENZ frequency, resistivity as a function of annealing vacuum level in the ITO films (I1–I3).

Reflection from ITO films was measured using variable angle spectroscopic ellipsometry (VASE, J.A. Woollam). ITO films were attached under a triangular prism using index-matching liquids for ATR measurements as shown in Fig. 1(a). To compensate the unwanted reflection from the prism surface, we performed the background measurement without the ITO films for each incident angle, and then calibrate the total reflection with the films using them.

Free carrier densities and ENZ frequencies in three ITO films (I1–I3) are determined by ellipsometry fitting to the Drude model (black and red curves), and are shown in Fig. 1(b). Drude model parameters are also obtained by Hall measurements (data not shown here), and they are found to agree with ellipsometry values. The ENZ frequency (*ω*_*ENZ*_) and the plasma frequency (*ω*_*p*_) in the Drude model are given by 
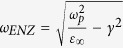
 and 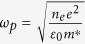, respectively. Here, *ε*_∞_ is the background dielectric constant at *ω* = ∞, *γ* is the damping constant of free electron, *n*_*e*_ is the free carrier density, m* ≈ 0.38 *m*_*e*_ is the effective mass of electron in conduction band (from Ref. [30]), and *ε*_0_ is the permittivity of free space. Carrier densities and ENZ frequencies decreases gradually in I1–I3, as the annealing vacuum level increases. The resistivity of the films was also obtained from the four-point probe measurement (blue curve). The resistivity increases gradually, as the free carrier density decreases in I1–I3.

The crystalline phase of ITO films was verified by X-ray diffraction (X’Pert-PRO MRD, Phillips), and all three ITO films (I1–I3) showed the strongest line at 2θ = 30.58° (Fig. S1, Supplementary Information), which corresponds to the reflection from the (222) crystalline plane of In_2_O_3_^33^,^34^.

**Figure 2:**
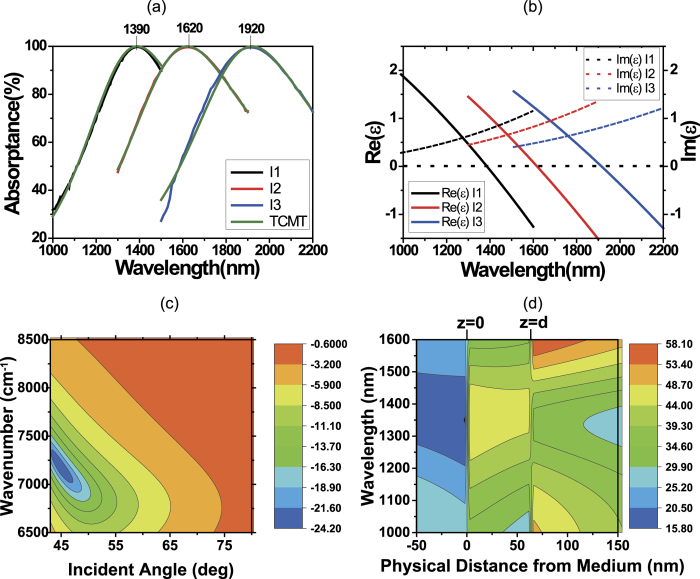
(**a**) Absorption spectra of three ITO films with different growth conditions. There is a gradual change in the PA wavelength. (**b**) The real (solid line) and imaginary (dotted line) parts of the dielectric constants of three ITO films that were measured by ellipsometry. There is a gradual change in the ENZ wavelength of the film. (**c**) The 10log(Reflectance) [dB] contour map as a function of frequency and incident angle is calculated by the transfer matrix method. Because *A* = 1 − *R* in our ATR configuration, *R* = −20 dB corresponds to *A* = 0.99. The black line is the ENZ mode dispersion curve. The PA point (white dot) lies on the ENZ mode dispersion. *ω*_*ENZ*_, which is determined from Re[ε(*ω*_*ENZ*_)] = 0, is drawn as a black broken line. (**d**) The magnitude of normal electric field (*E*_*z*_) in I1 is simulated at the PA wavelength (1390 nm), assuming the incidence angle of 46° and the incident irradiation of *I*_0_ = 1 W/m^2^.

The averaged atomic percentage of elemental composition (In, Sn, O) in ITO films (I1–I3) in Fig. S2 in SI was measured by the Auger electron spectroscopy (ESCA, K-Alpha, Thermo Scientific). The averaged elemental composition of indium (In) and tin (Sn) gradually decreases in the films I1–I3, while oxygen (O) increases. Especially the decrease of the Sn doping density is in agreement with Fig. 2(b), where the ENZ wavelengths gradually get longer in I1–I3. The red shift of ENZ wavelengths in I1–I3 can be explained as follows: as the annealing vacuum increases from 5 × 10^−6^ Torr (I1) to 5 × 10^−1^ Torr (I3), the oxidation process of ITO thin films is enhanced, and it limits the amount of Sn dopants or oxygen vacancies in ITO films. Therefore, the free carrier density in I1–I3 decreases, and thus the ENZ frequency decreases (or the ENZ wavelength increases).

## Results

Figure 1(a) shows a schematic of PA experiments in the ATR configuration studied in this paper. Previous radiative PA proposals^24^,^25^,^26^ assumed a metallic (or reflective) substrate to suppress transmission (*T* = 0), and determined the zero reflection condition. However, in the current work, we use the ATR configuration instead – i.e. we have a subwavelength ITO film under a prism and the substrate is air. This simplifies the sample fabrication and optical measurement in our case. This also eliminates unwanted, parasitic absorption in a metallic substrate.

### Tunable Perfect Absorption

We first demonstrate tunable PA using three different ITO films. Figure 2(a) shows the experimentally measured absorptance from these three samples. We obtained PA at different near-IR wavelengths at 1390 nm (I1), 1620 nm (I2), and 1920 nm (I3). The experimental PA spectra of the ITO thin films (I1–I3) were fitted by using the temporal coupled-mode theory (TCMT) [See S3 section in SI] and they are in good agreement in Fig. 2(a).

Figure 2(b) shows the real and imaginary parts of the dielectric constants (*Re*[ε], *Im*[ε]) of these ITO films as a function of wavelength, measured by variable angle spectroscopic ellipsometry^35^. Depending on the film deposition condition, there is a gradual change in the ENZ wavelengths, implying that doping densities vary gradually in the ITO samples. We find PA wavelengths located close to these ENZ wavelengths.

However, we notice that PA appears at slightly longer wavelengths than ENZ wavelengths (i.e. in the low frequency region). We theoretically studied this point in our previous paper^27^ and explained it in terms of the dispersion relation of ENZ modes^36^,^37^,^38^. Note that the ENZ mode is a bound mode (i.e. its dispersion lies on the right side of light line in air). But, because we are working in the ATR condition, we can access this bound mode. The reflectance *R* (in dB) of the ITO film I1 is calculated as a function of incident angle and frequency, using the transfer matrix method, and it is shown in Fig. 2(c). Because *A* = 1 − *R* in our ATR configuration, *R* = −20 dB corresponds to *A* = 0.99. The black dotted line in Fig. 2(c) is the ENZ frequency of the film I1. The black solid line is the dispersion relation of the ENZ mode existing in I1, and it lies slightly below its ENZ frequency. We compared the perfect absorption condition (incident angle, wavelength) with the ENZ mode dispersion, and found that the perfect absorption point (white dot in Fig. 2(c)) lies on the ENZ mode dispersion curve. Similar plots for the other two films (I2, I3) are given in Fig. S3 in SI, and they exhibit the same behavior.

The magnitude of the normal electric field (*E*_*z*_) in the film I1 is calculated in Fig. 2(d) as a function of the film depth (*z*) and the wavelength. The enhanced *E*_*z*_ field is observed around the ENZ wavelength. The uniform *E*_*z*_ field in the film is a typical characteristic of the ENZ mode. The film thickness (65 nm) is much less than the skin depth (~180 nm) of the ITO film I1. Similar field profile plots for the other two films (I2, I3) are given in Fig. S4 in SI, and the similar field enhancements are obtained near the ENZ wavelengths.

It was also pointed that the ENZ mode can appear only for small film thicknesses^39^ where the dispersion curve remains very close to the ENZ frequency. Although the film thicknesses in our work are slightly larger than those suggested in Ref. [39], the perfect absorption frequencies in our work are still very close to the ENZ frequencies *ω*_*ENZ*_ (within 90% of *ω*_*ENZ*_). And the electric field profile in the ITO film is highly flat near the perfect absorption frequency, as expected for the ENZ mode.

### Broadband Perfect Absorption

**Figure 3:**
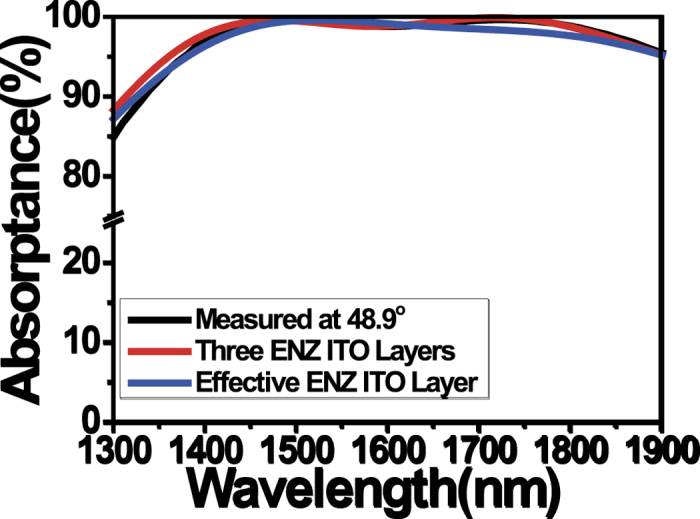
Experimentally measured absorption spectrum in the ITO multilayer film. The clear PA was achieved over a broad near-infrared wavelength range.

We have demonstrated highly tunable PA in the telecommunication window. However, this PA occurs at a single wavelength, and thus it is limited in use. To overcome this limitation, we deigned a multilayer of ITO films of different doping densities, based on our tunable ITO films in Fig. 2 and deposited it in RF magnetron sputtering. Figure 3 shows the measured absorptance from this ITO multilayer in the technologically important near-IR range. Absorption is close to 100% over a wide near-IR wavelength range of 1450 ~ 1750 nm at 48.9° incidence angle.

**Figure 4:**
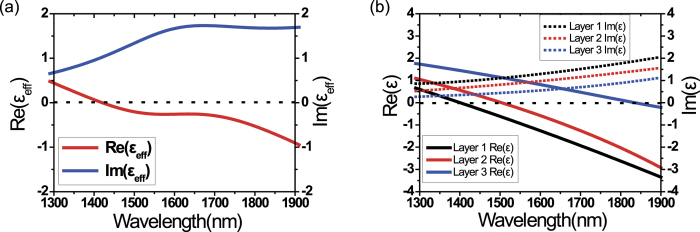
(**a**) Effective dielectric constant (ε_eff_) of the multilayer ITO films, obtained from the ellipsometry measurement. The real part of the effective dielectric constant (*Re*[ε_eff_]) is close to zero over the broad spectral region (i.e. broadband ENZ is achieved). (**b**) Modeling of the ITO multilayer as a three-layer system (Layer 1–3). The 2nd layer is an intermixed layer between two ITO films, formed during the annealing process. There is a gradual shift in the ENZ wavelength.

We obtained the dielectric constant of the ITO multilayer film using ellipsometry. Figure 4(a) shows the real and imaginary parts of the effective dielectric constant (ε_eff_) of the ITO multilayer (red and blue curves, respectively)^35^. The real part of the effective dielectric constant (*Re*[ε_eff_]) is close to zero over the broad spectral region (i.e. broadband ENZ is achieved). Using this effective dielectric constant, we could retrieve the broadband absorption spectrum in Fig. 3 (blue curve).

In fact, we notice that *Re*[ε_eff_] is slightly smaller than zero, rather than exactly zero. As discussed before, PA occurs at a slightly longer wavelength than the ENZ wavelength (Fig. 2). As shown in Fig. 2(b), *Re*[ε] decreases as the wavelength increases. Therefore, it is reasonable to have slightly negative values of *Re*[ε_eff_] at PA wavelengths, as shown in Fig. 4(a).

### Modeling of the ITO multilayer

To understand the optical response of the multilayer film more thoroughly, we also modeled the ITO multilayer as a three-layer system, including an intermixed layer between two ITO films. Figure 4(b) shows the dielectric constant of those three layers obtained from ellipsometry fitting^35^. We find that three layers (L1–L3) have different ENZ wavelengths and there is a gradual shift of the ENZ wavelength: 1400 nm (L1), 1503 nm (L2), and 1834 nm (L3). We also determined the effective thicknesses of three layers −52.2 nm (L1), 12.3 nm (L2), 58.5 nm (L3). The ellipsometry data were fitted using the Drude-Lorentz model to determine the material parameters of three layers:
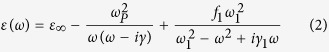
where *ε*_∞_ is the high frequency dielectric constant, *ω* is the angular frequency of incident light, *ω*_*p*_ is the plasma frequency, *γ* is the damping constant of free electrons, *γ*_1_ is the damping constant of bound electrons, and *f*_1_ is the electron oscillator strength. Table S1 in SI summarizes these Drude-Lorentz model parameters for each layer. We find that the electron density *n*_e_ of the 2nd layer (L2) is in between those of L1 and L3, as expected for an intermixed layer. The ENZ wavelength (1503 nm) of L2 is also in between those of two other layers (1400 nm (L1), 1834 nm (L3)). However, we see that the damping constant of L2 is bigger than the other two layers. These three-layer dielectric constants were again used to fit the broadband absorption spectrum in Fig. 3, and better fitting could be obtained (red curve).

## Discussion

**Figure 5:**
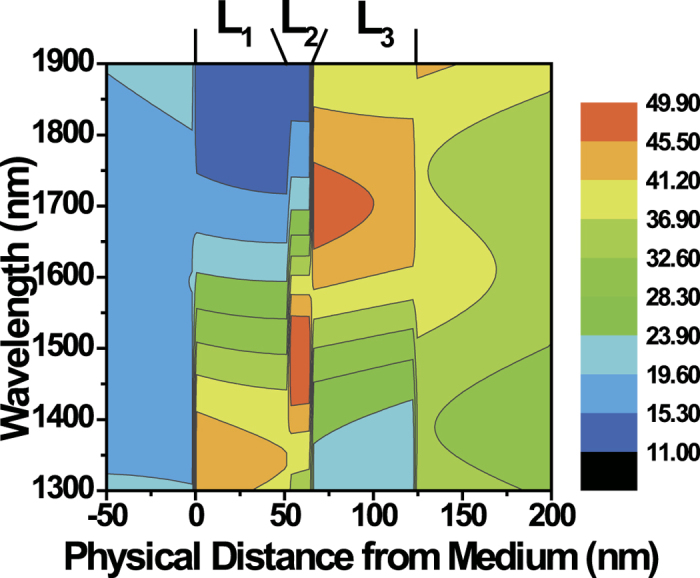
Contour map of the E_Z_ field distribution across the film as a function of wavelength (Color scale in V/m, assuming the incident irradiance of I_0_ = 1 W/m^2^). Each layer (L1–L3) has the field maximum at different wavelengths. The broadband field enhancement over the wide region of the near-IR spectrum results in broadband PA. The incident angle is 44°.

To further clarify the physical origin of broadband PA, we investigated the electric field distribution in the ITO multilayer using numerical simulations. The measured dielectric constants of ITO in Fig. 4(b) were used in numerical simulations. Figure 5 is the contour map of the E_Z_ field distribution across the film as a function of wavelength calculated by the transfer matrix method^40^. The displacement continuity condition (ε_1_E_1z_ = ε_2_E_2z_) leads to an interesting field distribution near an ENZ wavelength. When the dielectric constant ε becomes zero (i.e. ENZ), the corresponding normal electric field component (E_Z_) should diverge due to this boundary condition. But, in a real material, we have a small amount of the imaginary part of the dielectric constant though the real part is zero. However, we can still achieve reasonably high field enhancement at the ENZ wavelength (up to 50 V/m when the irradiance of incident light is 1 W/m^2^ in Fig. 5).

Because each ITO layer (L1–L3) has a different ENZ wavelength, the maximum field enhancement should occur at different wavelengths. Figure 5 verifies that the ITO layers L1–L3 have maximum field enhancement at different wavelengths as expected. The enhanced fields are shown in 1300–1400 nm in L1, 1400–1450 nm in L2, and 1650–1750 nm in L3, respectively. The gradual change of ENZ wavelengths in L1–L3 results in the gradual change of the field enhancement in the film. Therefore, this broadband field enhancement over the wide near-IR spectrum can lead to broadband PA that we have observed experimentally in Fig. 3. Though the E_Z_ field in L3 seems to decrease slightly across the film at 1700 nm, the actual variation of E_Z_ is less than ±2.5% of the center value, and the field profile remains almost constant across the film, as shown for a single ITO layer in Fig. 2(d).

**Figure 6:**
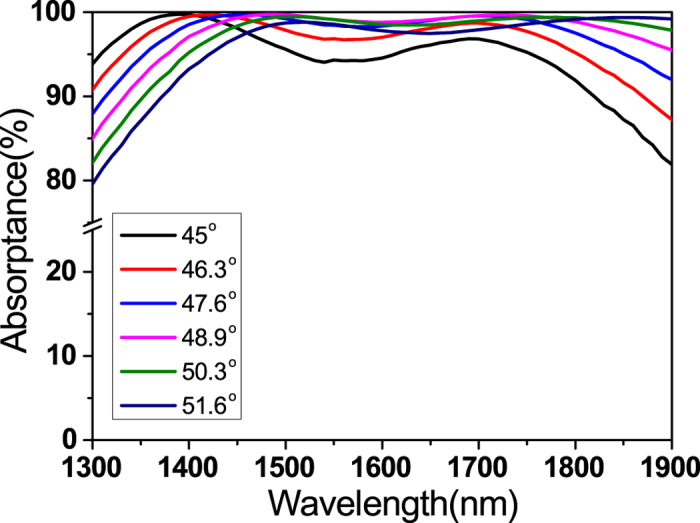
Angle dependence of broadband perfect absorption (PA) experimentally measured in the ITO multilayer. As the incident angle increases, the broadband PA shows a slight red-shift. Although the broadband PA is still angle-dependent, PA becomes more robust in a wide range of near-IR wavelengths due to its flat broadband nature.

Broadband PA relaxes the single wavelength condition in previous PA studies, and thus it is more suitable for many device applications. It also relaxes the incident angle dependence. PA in a single ENZ layer has the angle dependence given by Eq. (1). The angle dependence of light absorption in our ITO multilayer was experimentally measured in Fig. 6. As the incident angle increases, the broadband PA shows a slight red-shift. This can be explained by the redshift of ENZ mode dispersion (i.e. moving to a low frequency region) for larger incident angles (Fig. 2(c)). It is noted that though the broadband PA is still angle-dependent, PA becomes more robust in a wide range of near-IR wavelengths due to its flat broadband nature.

We further investigated the angle dependence by calculating the reflectance of the ITO multilayer film as a function of incident angle and frequency (Fig. S5, SI). Three reflection dips (or absorption peaks) are observed at ~6800 cm^−1^ (1470 nm) and 48° in L1, at ~5750 cm^−1^ (1740 nm) and 50° in L2, and at ~5250 cm^−1^ (1905 nm) and 54° in L3. In overall, a large area of PA can be found; e.g. the green region in Fig. S5 corresponds to larger than 99% absorption.

ITO is being largely used in the display and microelectronics industry as a transparent conducting material. Therefore, the use of ITO can facilitate the chip-scale integration of perfect absorbers with other device components. Moreover, the present work can be readily extended to doped semiconductors, of which ENZ wavelengths can cover the entire IR region. According to Kirchhoff’s law of thermal radiation, perfect absorbers should be perfect thermal emitters too^41^,^42^. Therefore, the current PA studies can be also applied to tunable and broadband thermal emitters. This can eventually lead to highly efficient, chip-scale thermal emitters in the IR region where efficient light sources are less readily available.

In summary, we realized tunable and broadband PA in the technologically important near-IR range. We used ITO films as near-IR ENZ materials, of which optical properties are highly tunable during the film growth. Tunable PA was demonstrated without any structural patterning. Moreover, using an ITO multilayer of different ENZ wavelengths, we realized broadband PA that covered a wide range of near-IR wavelengths. PA in a deep subwavelength volume is interesting itself from a fundamental point of view, and it can lead to highly efficient energy conversion. Especially, broadband PA relaxes the single wavelength condition in previous PA studies. Therefore, we believe that our work can make a significant contribution to important device applications that require efficient light absorption and energy conversion.

## Additional Information

**How to cite this article**: Yoon, J. *et al.* Broadband Epsilon-Near-Zero Perfect Absorption in the Near-Infrared. *Sci. Rep.* **5**, 12788; doi: 10.1038/srep12788 (2015).

## Supplementary Material

Supplementary Information
