# Supplementary material for: Broadband Epsilon-Near-Zero Perfect Absorption in the Near-Infrared
Source: Sci Rep. 2015 Aug 4;5:12788. doi: 10.1038/srep12788 (PMC4523933; doi:10.1038/srep12788)
Supplement: Supplementary Information [file srep12788-s1.pdf]

Supplementary Information

Broadband Epsilon-Near-Zero Perfect Absorption in the Near-Infrared

Junho Yoon[1], Ming Zhou[1], Md. Alamgir Badsha[1], Tae Young Kim[1], Young Chul Jun[2], Chang Kwon Hwangbo[1*]

[1]Department of Physics, Inha University, Incheon 402-751, Republic of Korea

[2]School of Materials Science and Engineering, UNIST, Ulsan 689-798, Republic of Korea

*hwangbo@inha.ac.kr

S1. XRD measurements (I1-I3)

S2. AES measurements (I1-I3)

S3. Resonant perfect absorption, contour map of 10Log(Reflectance) [dB], and dispersion of ITO films (I1 – I3)

S4. Contour map of normal electric field (E_z) of ITO films (I1 – I3)

S5. Contour map of 10Log(Reflectance) [dB] for the ITO multilayer (L1 – L3)

Table S1. Table for Drude-Lorentz model parameters of the ITO multilayer (L1 – L3)

References

S1. XRD measurements of ITO films (I1-I3)

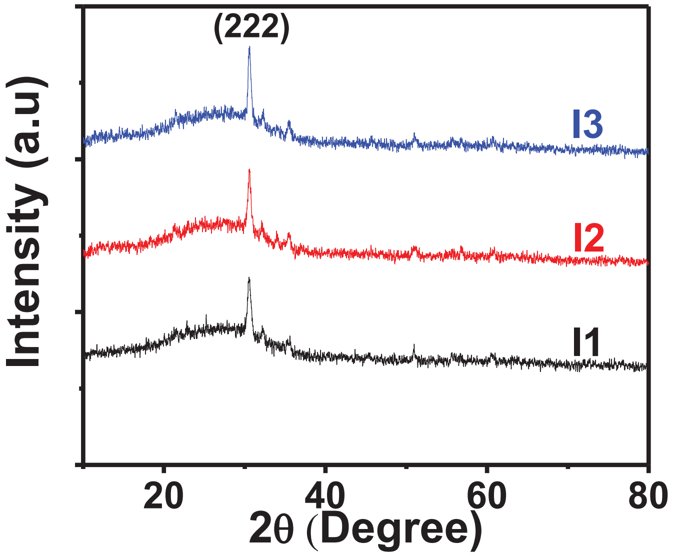

Figure S1. Crystallinity of ITO films (I1-I3)

The crystalline phase of ITO films was measured by X-ray diffraction. All three ITO films (I1-I3) showed the strongest line at $2\theta = 30.58°$ corresponding to the reflection from the (222) crystalline plane of In_2O_3, which verifies the crystallinity of ITO films. Without the annealing process, the film was amorphous In_2O_3.

S2. AES measurements of ITO films (I1-I3)

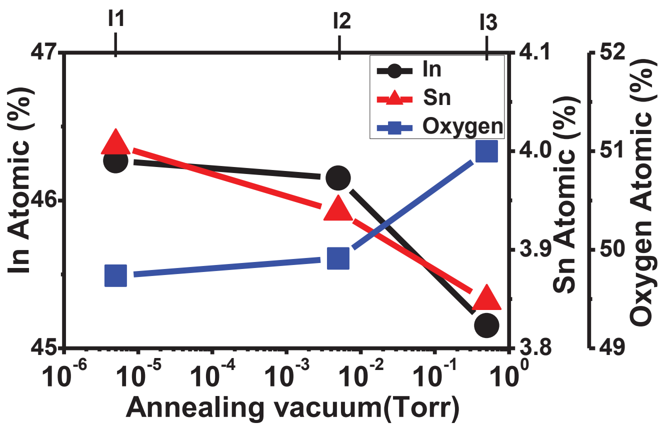

Figure S2. Averaged atomic percentage of elemental composition (In, Sn, O) in ITO films (I1-I3)

The averaged atomic percentage of elemental composition (In, Sn, O) in ITO films (I1-I3) was measured from the Auger electron spectroscopy (ESCA, K-Alpha, Thermo Scientific). The averaged elemental composition of indium (In) and tin (Sn) gradually decreases in the films I1-I3, while oxygen (O) increases. Especially the decrease of the Sn doping density in Fig. S2 is in agreement with Fig. 2(b), where the ENZ wavelengths gradually get longer in I1-I3.

S3. Resonant perfect absorption, contour map of 10Log(Reflectance) dB, and dispersion of ITO films (I1 – I3)

The resonant perfect absorption of three ITO films (I1-I3) was fitted by using the temporal coupled-mode theory [Ref. S1, S2]. For one-resonance and one-port system the absorptance $A(\omega)$ is given by

$$A(\omega) = \frac{4\Gamma_i\Gamma_r}{(\omega-\omega_{PA})^2+(\Gamma_i+\Gamma_r)^2} \tag{S1}$$

where $\omega_{PA}$is the PA frequency, Γ_i is the intrinsic damping constant of the system, and Γ_r is the radiative damping constant. Following the transfer matrix method the intrinsic and radiative damping constants are given by $\Gamma_i = \frac{1}{2}\omega_{PA}Im(\varepsilon)$ and $\Gamma_r = \frac{\pi d}{\lambda}\omega_{PA}n_0^3 \sin\theta_0 \tan\theta_0$, respectively, where $Im(\varepsilon)$ is the imaginary part of the dielectric constant of ITO film, d is the thickness, and θ_0 is the incident angle in the prism [Ref. S3]. We find in Eq. (S1) that the PA ($A = 1$) at the peak frequency ω_{PA} occurs at Γ_i=Γ_r. We obtained $\Gamma_{i1} = \Gamma_{r1} = 1.69 \times 10^{14}$ [rad/s] for I1, $\Gamma_{i2} = \Gamma_{r2} = 1.40 \times 10^{14}$ [rad/s] for I2, and $\Gamma_{i3} = \Gamma_{r3} = 1.03 \times 10^{14}$ [rad/s] by fitting Eq. (2) to the experimental spectrum. Figure 2(a) shows that the experimental PA spectra match well with the temporal coupled-mode theory.

(a) I1

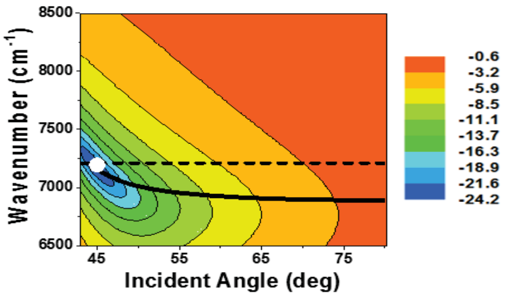

(b) I2

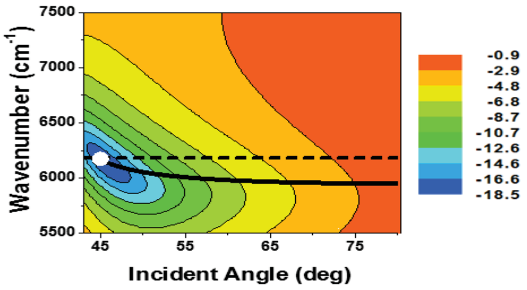

(c) I3

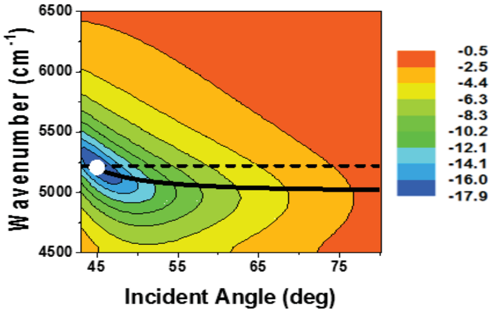

Fig. S3. Contour map of 10Log(Reflectance) [dB] and dispersion of ITO films ((a) I1, (b) I2, (c) I3)

The 10log(Reflectance) [dB] of the ITO film (I1-I3) as a function of incident angle and frequency is calculated using the transfer matrix method. Because A = 1 – R in our ATR configuration, R = -20 dB

corresponds to A = 0.99. The black dotted line is the ENZ frequency in wavenumber (cm^{-1}) in the ITO film. The black solid line is the dispersion relation of the ENZ mode existing in the ITO film, and it lies slightly below its ENZ frequency. The ENZ mode lies around the epsilon zero frequency for a small wavevector region, but the mode dispersion slightly redshifts (i.e. moves to a low frequency region) and becomes nearly flat for larger wavevectors. The PA point (white dot) lies on the ENZ mode dispersion, and thus the PA frequency is located slightly below the epsilon zero frequency ($Re[\varepsilon] = 0$), in agreement with our experiment data (Fig. 2(a),(b)).

S4. Contour map of normal electric field (E_z) of ITO films (I1 – I3)

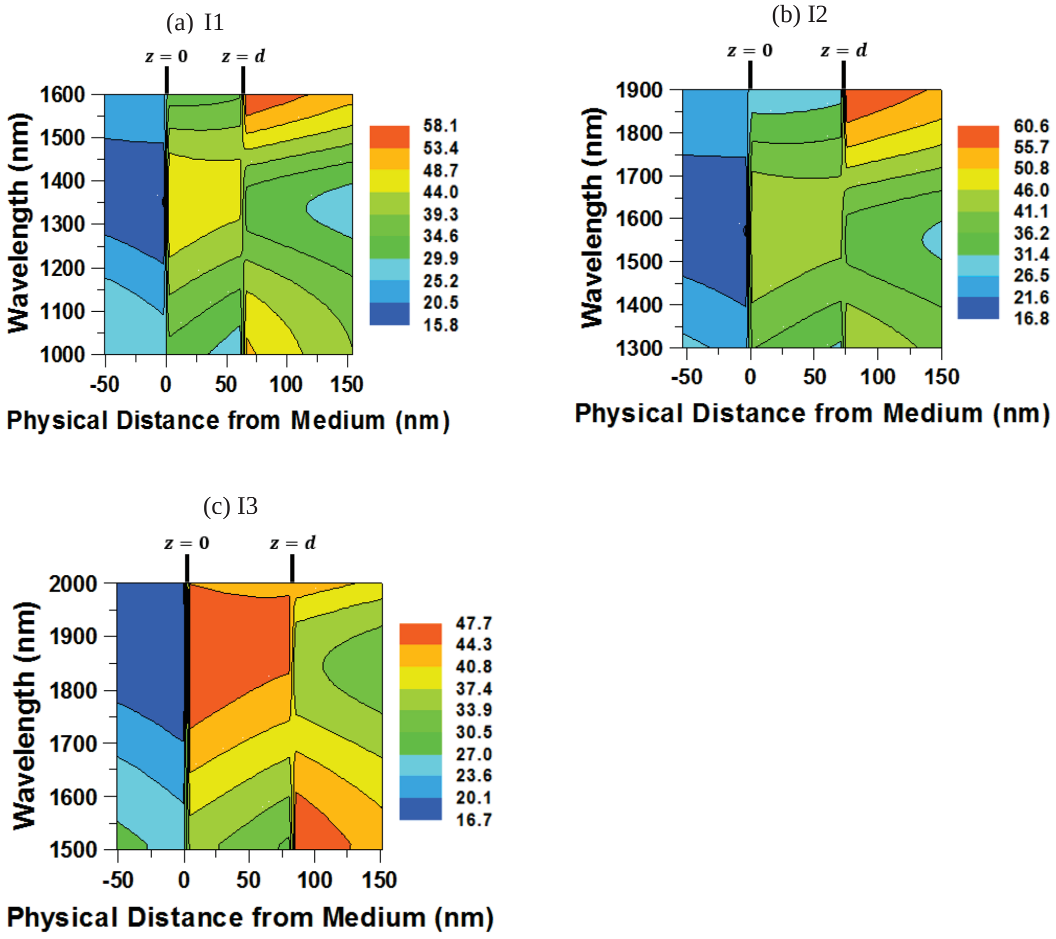

Fig. S4. Contour map of normal electric field (E_z) of ITO films (I1 – I3)

The magnitude of the normal electric field (E_z) of the ITO films (I1 – I3) is calculated as a function of the depth of the film (z) and the wavelength (incidence angle: 46°, incident irradiation of $I_0 = 1\ \mathrm{W/m^2}$). The uniformly enhanced fields (E_z) of the ITO films (I1 – I3) are shown at different ENZ wavelength regimes ((a) 1390 nm, (b) 1620 nm, and (c) 1920 nm, respectively).

S5. Contour map of 10Log(Reflectance) dB for an ITO multilayer (L1-L3)

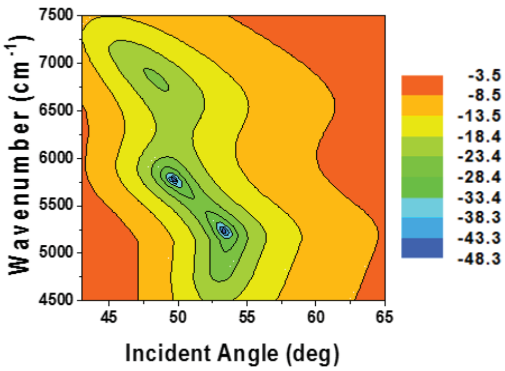

Fig. S5. Contour map of 10Log(Reflectance) [dB]

The 10Log(Reflectance) [dB] contour map of the ITO multilayer is calculated as a function of incident angle and frequency. Because A = 1 – R in our ATR configuration, R = -20 dB corresponds to A = 0.99. Three reflection dips (or absorption peaks) are shown together with a broad region of perfect absorption.

Table S1. Table for Drude-Lorentz model parameters of broadband ITO multilayer

	Layer 1	Layer 2	Layer 3
$\omega_p(10^{15} rad/s)$	2.7984	2.1523	1.8139
$\gamma(10^{14} rad/s)$	2.7031	3.7766	1.7046
$n_e(10^{20}/cm^3)$	9.3662	5.5402	3.9350
$\rho(10^{-4}\Omega \cdot cm)$	3.8984	9.2078	5.8516
f_1	0.1333	2.5930	0.4605
$\omega_1(10^{15} rad/s)$	1.7374	0.7113	0.8418
$\gamma_1(10^{14} rad/s)$	0.3656	0.0016	3.4989

Table S1. Drude-Lorentz model parameters of three layers (L1-L3), obtained from ellipsometry fitting.

References

S1. Haus , H. A., Waves and Fields in Optoelectronics, Prentice-Hall, Englewood Cliffs, N.J. (1984).

S2. Fan, S., Suh, W. & Joannopoulos, J. D., Temporal coupled mode theory for Fano resonance in optical resonators, *J. Opt. Soc. Am. A*, **20**, 569–572 (2003).

S3. Badsha, M. A., Jun, Y. C. & Hwangbo, C. K. Admittance matching analysis of perfect absorption in ultra-thin, unpatterned films. *Opt. Commun.* **332**, 206-213 (2014).
